# The Burden of the Past: A Systematic Review of Childhood Trauma and Mental Health in Transgender and Gender Nonconforming Individuals

**DOI:** 10.3390/ejihpe15090183

**Published:** 2025-09-12

**Authors:** Giulia Di Fini, Cristina Civilotti, Annalisa Bolognino, Gabriele Einaudi, Mariateresa Molo, Fabio Veglia, Gabriella Gandino, Sarah Finzi

**Affiliations:** 1 Department of Psychology, University of Turin, 10124 Torino, Italy; 2 Centro Clinico Crocetta, Scuola di Specializzazione in Psicoterapia Cognitiva, 10129 Turin, Italy; 3 Carlo Molo Foundation, 10123 Turin, Italy

**Keywords:** adverse childhood experiences (ACEs), transgender and gender nonconforming (TGNC), mental health, trauma-informed care, minority stress, victimization

## Abstract

Adverse childhood experiences (ACEs) are critical determinants of long-term psychological and physiological health outcomes. Transgender and gender nonconforming (TGNC) individuals are at increased risk for ACEs, including family rejection, peer victimization, and systemic discrimination. Despite the growing body of research on this topic, an updated synthesis of recent literature is needed to understand the evolving landscape of ACE-related risks and protective factors in TGNC populations. This systematic review, conducted according to PRISMA guidelines, examined studies published between 2020 and 2024 that were related to the association between ACEs and mental health outcomes in TGNC individuals. A comprehensive database search yielded 6124 articles, 42 of which met the inclusion criteria. Data extraction focused on the type of ACEs reported, associated mental health outcomes, resilience factors, and clinical implications. The results showed that TGNC individuals are significantly more likely to experience childhood maltreatment, including emotional, physical, and sexual abuse, as well as transphobia-specific ACEs such as forced gender conformity and identity denial. These experiences are associated with an increased risk of depression, PTSD, suicidality, and substance use disorders. Family rejection was found to be a critical risk factor, while social support, gender-specific care, and self-efficacy showed protective effects. The reviewed studies emphasize the urgent need for trauma-informed and gender-affirming mental health interventions to mitigate the effects of ACEs on TGNC individuals. Limitations and future research directions are discussed.

## 1. Introduction

Transgender and gender nonconforming (TGNC) people are people whose gender identity does not align with the sex assigned to them at birth and/or who fall outside the traditional gender binary. These identities, which encompass a wide range of experiences, make it clear that living with a gender outside of conventional norms brings with it particular challenges on a social, relational, and institutional level.

TGNC individuals appear to be disproportionately affected by early traumatic experiences, often triggered by familial rejection, systemic discrimination, and social stigmatization.

The concept of adverse childhood experiences (ACEs) refers to a range of traumatic events in childhood, including physical, emotional, and sexual abuse, neglect, and a dysfunctional family environment (Felitti et al., 1998). Numerous studies have shown that the accumulation of such experiences increases both psychological and physical vulnerability and predisposes individuals to long-term negative outcomes such as depressive disorders, anxiety, and other mental health problems (Bick & Nelson, 2016; Danese et al., 2009).

Research consistently shows that ACEs are more prevalent in TGNC individuals compared to cisgender populations. For example, 93% of TGNC participants in one study reported at least one adverse childhood experience, with 30% experiencing severe to extreme forms of abuse (Biedermann et al., 2021; Feil et al., 2023). In addition, the negative effects of discrimination on mental health have been studied (Keating & Muller, 2020), and hostile environments, stigmatization, experiences of violence, and rejection have been shown to expose people to chronic traumatic stress, referred to as the minority stress model (Meyer, 2003).

Given the increasing visibility of TGNC individuals in both research and clinical practice, there is an urgent need to update and expand the existing knowledge base on the impact of ACEs in this population. Building on studies published between 2020 and 2023, this systematic review seeks to synthesize current evidence and critically examine how early adverse experiences affect TGNC people across psychological and physical domains.

Building on earlier systematic reviews that covered studies prior to 2020, this review deliberately focuses on research published between 2020 and 2023. This time window was chosen for two main reasons: first, because the scientific literature on ACEs in TGNC populations has expanded rapidly in the past few years, with a proliferation of heterogeneous studies that require an updated synthesis; and second, because recent studies have begun to address new aspects—such as nonbinary experiences, the role of online environments, and intersectional factors—that were largely absent from earlier reviews. By concentrating on this period, this review aims to provide a structured and up-to-date overview of the most recent evidence in a rapidly evolving field.

To guide the review, we formulated the following research questions:

(1) What is the prevalence and typology of adverse childhood experiences reported by TGNC individuals in recent empirical studies?
(2) How are these experiences associated with mental health and physical health outcomes, and to what extent do resilience and protective factors (e.g., gender-affirming care, social support) moderate these associations?
(3) Which conceptual and methodological gaps remain in the literature, and what directions are most promising for future research, including longitudinal and intersectional approaches?

These guiding questions anchor the subsequent analysis and structure the presentation of results and discussion.

## 2. Materials and Methods

This systematic review was conducted following the guidelines provided by the PRISMA (Preferred Reporting Items for Systematic Reviews and Meta-Analyses) statement (Page et al., 2021). The review protocol is available in the International Prospective Register of Systematic Reviews (PROSPERO) under the identifier CRD42023483493.

### 2.1. Search Strategy

A systematic search of the literature was conducted in four different scholarly databases: PubMed, Web of Science, PsycInfo, and PsycArticles. The search was restricted to articles focused on trauma in the TGNC population and was limited to publications in English between 1 January 2020 and 31 December 2024. The selection of keywords was guided by an initial scoping review conducted during the preliminary phase of the study, before performing the comprehensive database search. Following this initial scoping review, the high number and heterogeneity of studies identified prompted us to narrow our focus and conduct a systematic review specifically on adverse childhood experiences in TGNC individuals. This decision aimed to provide a more structured and in-depth synthesis of the literature on early-life trauma, allowing for a clearer understanding of its long-term mental health consequences and the interplay of risk and protective factors within this population.

The keywords employed in the four databases are detailed in Table 1. Three authors independently conducted research across the four databases using specific keywords and subsequently applied the following filters: peer-reviewed, academic publications, empirical studies, English language, human samples, and exclusion of dissertations. These filters were adapted and modified according to the advanced search features of each specific database. Therefore, papers that were not peer-reviewed, not academic publications, not empirical studies, not in English, not based on human samples, or that were dissertations were excluded.

A total of 6124 records were identified through database searching; after removing duplicates and applying the filters, 2830 papers remained.

### 2.2. Eligibility Criteria and Selection Process

Papers had to meet the following criteria to be included: (a) including child, adolescent, and/or adult TGNC participants (studies where TGNC participants were not analyzed separately but were grouped with non-TGNB populations (e.g., sexual minorities) were also excluded); this criterion was applied to focus the synthesis on the specific developmental, psychosocial, and clinical experiences of TGNC populations, which may be obscured when data are pooled with those of sexual minority groups; (b) addressing well-documented early traumatic experiences (in terms of mental health effects, epidemiological analysis, or implications for clinical interventions; studies that solely mentioned traumatic experiences among the risk factors for mental health issues or reported traumatic histories beginning in adulthood were excluded); (c) not being reviews, practice guidelines, theoretical essays, editorials, letters to the editor, or conference proceedings; and (d) not being single-case studies. Both qualitative and quantitative studies were included.

**Figure 1:**
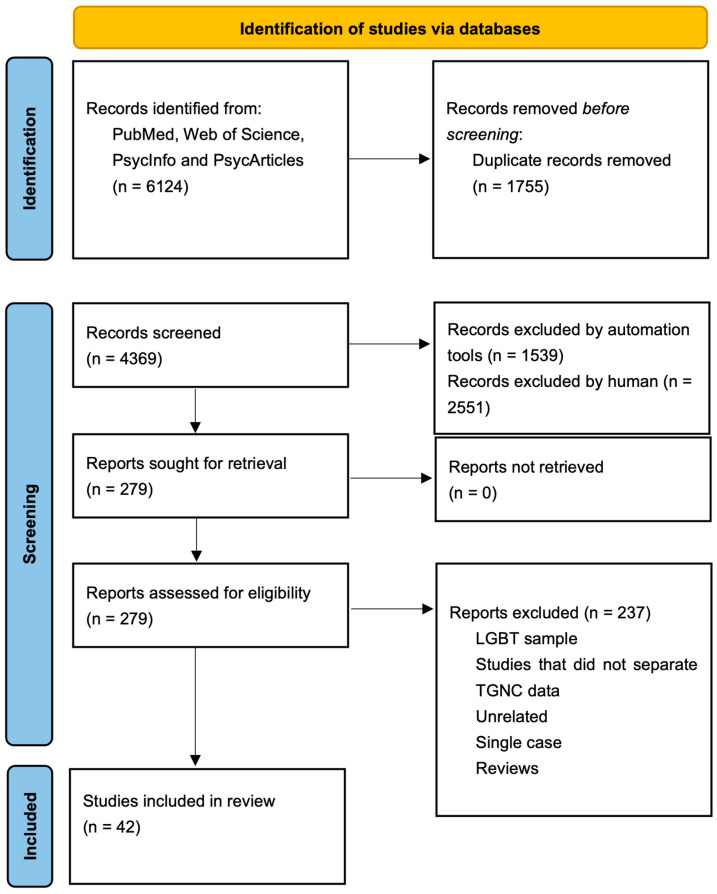
PRISMA flow diagram.

The screening process based on titles and abstracts identified 279 papers for eligibility (2551 were excluded), and the final selection included 42 studies that met the inclusion criteria (237 were excluded). Figure 1 illustrates the selection process for this systematic review; the included papers are summarized in Table 2.

### 2.3. Data Extraction and Synthesis

For each included study, descriptive information was systematically extracted and organized in a Microsoft Excel spreadsheet developed specifically for this review. A coding framework, developed a priori based on the research questions, guided the extraction process. The following information was extracted from each study (Table 2): (a) first author, year, and country; (b) sample characteristics (sample size, age range/mean age, sex assigned at birth); (c) type of traumatic experiences studied; (d) study design and methodological approach; (e) health implications; and (f) clinical implications derived from the findings.

Data extraction and initial coding were conducted by two reviewers, with any ambiguities discussed in collaboration with the other authors to ensure accuracy and consistency. Although we did not calculate formal interrater reliability coefficients (e.g., Cohen’s κ), the process was structured to maximize transparency and agreement. Once coding was complete, a comprehensive Excel spreadsheet was created summarizing all included studies and relevant predictors. One reviewer categorized the reported traumatic experiences into the standard ACE domains, and two additional reviewers independently checked these classifications. Disagreements were resolved through discussion until a consensus was reached.

During the screening and selection phase, all titles, abstracts, and full texts were independently assessed by two reviewers. Disagreements regarding eligibility were first discussed between the two reviewers, and unresolved cases were referred to a third lead reviewer, whose decision was considered final. This multi-stage process ensured that inclusion and exclusion decisions were rigorous and reproducible.

All studies included in the systematic review were synthesized and summarized narratively.

Given the heterogeneity of study designs, populations, and outcome measures, a narrative synthesis approach was adopted. The quantitative results (e.g., prevalence estimates, effect sizes, and associations) were summarized descriptively, while the qualitative results were examined thematically. To integrate these two forms of evidence, we applied a convergent synthesis logic: findings from qualitative studies were used to contextualize and deepen the interpretation of quantitative patterns, and areas of convergence and divergence were explicitly identified.

The synthesis process comprised three iterative steps. First, the extracted data were grouped according to the research questions (prevalence and typology of ACEs, associations with mental and physical health, and protective factors). Second, the results within each area were organized by study design (quantitative or qualitative) to allow comparison between different paradigms. Third, overarching themes were developed inductively through repeated discussions among reviewers, focusing on patterns that were consistently confirmed across multiple studies.

Our synthesis was guided by a pragmatic epistemological approach that emphasizes the integration of different forms of evidence to identify clinical and research implications, rather than the primacy of a single methodological paradigm. This approach enabled us to highlight robust results despite the methodological and cultural diversity of the included studies.

A meta-analysis could not be performed due to the heterogeneity of the results, populations, or study designs.

### 2.4. Quality Assessment

In this systematic review, a total of 38 quantitative and 4 qualitative studies were evaluated for their methodological quality. Among the 38 analytical cross-sectional studies assessed using the JBI Checklist for Analytical Cross-Sectional Studies (Joanna Briggs Institute, 2017), the majority demonstrated high methodological rigor. More specifically, 25 studies (65.8%) met at least 7 of the 8 criteria and were rated as high quality. These studies generally had clearly defined inclusion criteria, standardized outcome measures, and used appropriate statistical analyses. Twelve studies (31.6%) were classified as moderate quality and met 5 to 6 criteria. Methodological limitations in this group were often related to the identification and handling of confounding variables and the use of self-report measures with limited psychometric validation. One study (2.6%) was rated as low quality because it met fewer than five criteria and had several significant design flaws. All four qualitative studies were assessed using the JBI Checklist for Qualitative Research (Lockwood et al., 2015) and were rated as high quality because they each met between 8 and 10 of the 10 criteria. These studies consistently demonstrated consistency between their epistemological stance and chosen methodology, conducted rigorous data analysis, and adhered to sound ethical research practices. Nevertheless, minor limitations were noted, particularly in relation to the transparency of the researchers’ positionality and reflexivity. For example, one study did not explicitly discuss how the researchers’ perspective may have influenced the study design and interpretation.

Overall, the quality assessment indicates that the evidence base contained in this review is largely sound in methodological terms. The quantitative studies were, for the most part, well designed and well conducted, while the qualitative studies met the high standards of qualitative research. Future research could further improve methodological transparency through a more systematic inclusion of reflective practices in qualitative research.

## 3. Results

The 42 studies included in this review are based on data collected between 2005 and 2021. Most of them were conducted in the Americas: 24 studies in the United States, 2 in Canada, and 1 in Mexico. In addition, six studies are from Europe. The paper also includes three studies from China, three studies from Australia, and one study each from Thailand and India.

To facilitate an analytical synthesis, the results are divided into two broad categories: (A) traditional ACEs, which include forms of mistreatment and neglect that are not specific to gender identity, and (B) TGNC-specific ACEs, which include identity-based rejection, systemic discrimination, and minority stress. Within each category, results are further grouped by key themes inspired by the Minority Stress Model (Meyer, 2003): distal stressors (structural and communal factors), proximal stressors (familial rejection, peer victimization, internalized stigma), and resilience/protective mechanisms.

### 3.1. Traditional ACEs

#### 3.1.1. Prevalence of Traditional ACEs

Research underscores the high prevalence and diversity of traumatic experiences in TGNC individuals. For example, Biedermann et al. (2021) found that 93% of TGNC respondents had at least mild to moderate childhood adverse experiences, while 30.2% had severe to extreme experiences. Newcomb et al. (2020) reported that nonbinary assigned-male-at-birth (AMAB) youth had the highest rates of lifetime trauma (82.1%), followed by nonbinary assigned-female-at-birth (AFAB) youth (78%), trans women (72.4%), and trans men (59.1%) (see Table 2 for sample size). Similarly, Suarez et al. (2021) found that 92% of trans men reported adverse childhood experiences (ACEs), while Belloir et al. (2023) found that 42.2% of participants had experienced four or more ACEs. Several studies confirm that TGNC individuals are significantly more likely to experience adverse childhood experiences compared to their cisgender peers (Brangwin et al., 2023; Feil et al., 2023; Kaltiala & Ellonen, 2022; Kozlowska et al., 2021; Lian et al., 2022; Thoma et al., 2021; Tran et al., 2023; Wichaidit et al., 2021). These experiences vary widely and include childhood maltreatment, bullying, sexual abuse, systemic discrimination, and community violence. TGNC respondents frequently reported parental violence (23.6%) or stated that their parents regularly forced them to conform to their sex assigned at birth (25.7%) (Biedermann et al., 2021). Physical abuse was more often committed by a family member (24.8%) than by someone outside the family (16.2%), while other forms of familial abuse, such as neglect and emotional or verbal abuse, were reported by 57.9% of participants (Strauss et al., 2020). In contrast, sexual abuse was more often perpetrated by someone outside the family (24.3%) than by a family member (7.5%). Childhood sexual abuse (CSA) is a common problem among TGNC individuals. Xu et al. (2023) found that 14.2% of trans women in China reported CSA, while higher rates were observed in studies by Ricks and Horan (2023) (40.6%) and Newcomb et al. (2020) (more than one-third of trans women and nonbinary AMAB individuals). In the Eastwood et al. (2021) and Tubman et al. (2024) samples, approximately 50% of participants had experienced CSA.

#### 3.1.2. Effects of Traditional ACEs on Mental and Physical Health

The studies included in this review consistently report a link between general adverse childhood experiences and an increased risk of mental health disorders, including depression, anxiety, posttraumatic stress disorder (PTSD), and suicidality. Biedermann et al. (2021) found that ACEs were strongly associated with increased rates of depression and suicidality in adulthood, emphasizing the long-term psychological burden of early trauma. Suarez et al. (2021) focused specifically on AFAB adults and found that over 90% reported at least one ACE, and those with four or more ACEs were at significantly higher risk for depression, suicidality, PTSD, and intimate partner violence. Several studies document the alarming prevalence of suicidal ideation and attempts among TGNC individuals with a history of ACEs. Brangwin et al. (2023) found a significant link between family-based physical abuse and suicide attempts in gender minority adolescents. In addition, Domínguez-Martínez et al. (2023) identified psychological violence, common in family and school settings, as a key predictor of suicidality in transgender individuals. Garthe et al. (2022) found that peer victimization and depressive symptoms were strong mediators in the relationship between gender identity and suicidal ideation, highlighting the compounding effects of early social rejection. Thoma et al. (2021) demonstrated that transgender adolescents were significantly more likely to experience psychological, physical, and sexual abuse compared to their cisgender peers, increasing their suicide risk. Cao et al. (2023) provided further insights into the mechanisms underlying self-injurious behavior in TGNC individuals. In their study, a strong association was found between childhood abuse and non-suicidal self-injury (NSSI) mediated by emotion dysregulation traits. The risk of lifetime suicide attempts (LSA) was found to be higher among transgender individuals, particularly transgender men, compared to cisgender individuals. ACEs increase LSA risk for all groups, but the effect is stronger for transgender populations (Barboza-Salerno & Meshelemiah, 2024). Depressive symptoms mediate this link, with gender identity moderating the effect.

Beyond mental health, traditional ACEs also have significant effects on physical health. Dysregulation of the hypothalamic–pituitary–adrenal (HPA) axis due to prolonged stress exposure underlies many chronic conditions in TGNC individuals. These include obesity, cardiovascular disease, and autoimmune diseases (Marquez-Velarde et al., 2023). Childhood sexual abuse disproportionately affects TGNC individuals, which has a long-term impact on sexual health. They are more likely to engage in high-risk sexual behavior, such as transactional sex, which in turn increases the risk of sexually transmitted infections, including HIV (Suarez et al., 2021).

#### 3.1.3. Protective and Mediating Factors

Despite the severe psychological consequences of ACEs, some studies point to potential protective factors that can mitigate these effects. Chakrapani et al. (2022) emphasized the role of resilience strategies such as community support and a gender-affirming environment in reducing the psychological distress of trans AFAB individuals. In addition, Ricks & Horan, 2023) examined the role of gender affirmation on mental health and found that strong social affirmation and support networks serve as critical protective factors against depression, anxiety, body image dissatisfaction, and self-harm in Black transgender women; however, intimate partner violence interferes with the positive effects of gender affirmation on quality of life and anxiety symptoms, which is consistent with findings in psychotraumatology. Thomeer et al. (2022) examined the lived experiences of transgender and nonbinary individuals and found that community inclusion and gender exploration are important elements in promoting resilience.

### 3.2. TGNC-Specific ACEs

TGNC-specific ACEs refer to identity-related experiences of rejection, discrimination, and systemic barriers that go beyond traditional definitions of childhood adversity. These factors clearly emerge in the literature reviewed and can be directly mapped to the proximal and distal stressors of the Minority Stress Model (Meyer, 2003).

#### 3.2.1. Identity-Related Rejection in the Family and Among Peers

TGNC respondents frequently reported that their parents regularly forced them to conform to their sex assigned at birth (25.7%) (Biedermann et al., 2021). Transfeminine individuals were more likely to report emotional and physical abuse, while transmasculine individuals were more likely to report efforts to change their identity and expression. Family stressors—such as non-acceptance, marriage, and reproductive pressures—were associated with non-disclosure of gender identity, suppression of gender expression, and family violence (Chan et al., 2024).

Family rejection not only isolates TGNC adolescents but also leads to chronic shame, resulting in persistent feelings of unworthiness and insecurity (Strauss et al., 2020; Garthe et al., 2022). The lack of parental mirroring of the child’s identity can further exacerbate identity fragmentation and increase vulnerability to external sources of harm (Ricks & Horan, 2023).

TGNC youth are disproportionately affected by bullying, which leads to cycles of trauma. They experience more violence at school, have more safety concerns, and are more likely to skip school than gender-conforming peers (Klemmer et al., 2021). TGNC students are also more prone to traditional and cyberbullying (Lian et al., 2022). In addition, 89% of transgender youth report rejection by peers, often at school, leading to greater social isolation (Strauss et al., 2019). High levels of gender nonconformity further increase peer victimization (van Beusekom et al., 2020).

#### 3.2.2. Systemic and Structural Discrimination

Structural and interpersonal discrimination, including school victimization and peer harassment, create additional layers of risk that extend beyond childhood into adolescence and adulthood (Lian et al., 2022; Klemmer et al., 2021). Such experiences not only increase minority stress but also contribute to a higher likelihood of substance use and participation in risky behaviors as maladaptive coping strategies (Newcomb et al., 2020; Lowry et al., 2020). In the school context, TGNC youth are often not protected from bullying and harassment, which exacerbates feelings of alienation and distress (Xu et al., 2023; Feil et al., 2023). In healthcare, a lack of training in gender-equitable care leads to stigmatization and denial of services, which exacerbates inequalities in physical and mental health (Cao et al., 2023; Garthe et al., 2022).

Societal stigmatization of TGNC individuals, coupled with systemic failures to provide adequate protection or resources, leaves many without the means to recognize or escape these harmful dynamics, exacerbating their long-term mental and social distress (Sizemore et al., 2022; Feil et al., 2023).

Discriminatory hiring practices and systemic exclusion force many TGNC individuals into precarious labor markets; one example is sex work, where they are at greater risk of violence and exploitation (Ricks & Horan, 2023; Sizemore et al., 2022). Economic instability limits access to resources needed for recovery, such as safe housing and healthcare, which exacerbates the cycle of trauma and hardship (Xu et al., 2023).

Internalized transphobia and minority stress create a self-reinforcing cycle of hypervigilance and avoidance in which TGNC individuals withdraw from potential sources of support for fear of discrimination or rejection. This isolation deprives them of protective factors, such as community connections and affirming relationships, which are crucial for mitigating the effects of trauma (Feil et al., 2023; Cao et al., 2023).

#### 3.2.3. Trauma in the Context of Gender Nonconformity

In the various articles reviewed, authors have emphasized that gender nonconformity in different contexts is an element of vulnerability that contributes to the occurrence of traumatic experiences that severely affect the mental health and general well-being of the individuals concerned. In particular, many studies show that transgender and gender nonconforming people are highly victimized in both familial and social contexts due to the rejection of their gender identity by others. Some studies show that negative reactions to gender reassignment can have a significant impact on the mental health and psychological well-being of those affected (Cussino et al., 2021). In several cases, family rejection manifests itself in direct violence, both physical and psychological, leading to greater vulnerability to depression and anxiety (Domínguez-Martínez et al., 2023). In addition to the family dimension, the trauma associated with gender nonconformity manifests itself with particular intensity in the school environment and among peers. Many gender-atypical adolescents report experiences of exclusion, bullying, and verbal and physical violence, which have serious psychological consequences, including an increased risk of self-harm and suicidal thoughts (van Beusekom et al., 2020; Garthe et al., 2022). Some studies suggest that gender nonconformity is closely associated with cyber-victimization and contributes to feelings of isolation (Lian et al., 2022). High levels of microaggressions, community rejection of gender expression leading to severe emotional distress, and, in the most extreme cases, the need to hide or suppress one’s gender identity to avoid experiences of violence are also noted (Austin et al., 2022; Chakrapani et al., 2022). Studies show that the censorship and social pressure associated with gender reassignment can have a profound psychological impact, affecting the general well-being of those affected and contributing to their psychosocial distress (Kozlowska et al., 2021). The rigidity of gender norms, which often permeate the family and school context, is used as an instrument of coercion and control (Kaltiala & Ellonen, 2022). Sometimes sexual harassment is used as a means of punishing or correcting non-compliant gender expression, which exacerbates the trauma of those affected (M. A. Price et al., 2023).

#### 3.2.4. Structural and Communal Trauma

Consistent with Minority Stress Theory (Meyer, 2003), distal stressors such as structural and communal trauma are a central aspect of the lived experiences of many TGNC individuals. The studies examined show how institutional and social discrimination contribute significantly to the marginalization and vulnerability of these people. A recurring theme in the studies is the lack of legal protections and adequate measures to ensure the safety and well-being of TGNC people, particularly in the workplace and healthcare settings (Chakrapani et al., 2022). Institutional barriers limit access to essential services and increase the risk of social exclusion and poverty (Newcomb et al., 2020). One particularly obvious aspect is the homelessness of transgender people, which is frequently a direct result of family rejection and social discrimination (Eastwood et al., 2021). Some studies report that these people face significant risks due to economic hardship and lack of social support, including the need to engage in dangerous activities such as forced sex work in order to survive (Suarez et al., 2021). In addition, school and work policies often do not include specific protections for gender nonconforming people, increasing the risk of violence and structural discrimination (McBride & Neary, 2021). Structural trauma is also evident in the lack of access to adequate health services. Many studies show how transgender people are discriminated against in medical facilities, which has serious consequences for their mental and physical health (Lacombe-Duncan & Olawale, 2022). Stigmatization in the healthcare system often results in people seeking less care and treatment, which has a negative impact on their quality of life and general well-being (Ricks & Horan, 2023). The school context also plays a key role in perpetuating structural trauma. The school environment often does not provide adequate support for transgender and gender nonconforming students, contributing to bullying and systematic victimization (Klemmer et al., 2021). TGNC individuals are also more likely to experience violence at the hands of law enforcement, which exacerbates feelings of insecurity and distrust of institutions (Strauss et al., 2019).

#### 3.2.5. Effects of TGNC-Specific ACEs on Mental Health

Other studies emphasize the consequences of ACEs that are directly linked to gender identity and minority stress. In studies examining PTSD in TGNC individuals, Arayasirikul et al. (2022) documented that childhood transphobic adversity was widespread in young transgender women, with high rates of PTSD and other trauma-related disorders. Similarly, van Beusekom et al. (2020) reported that peer victimization was associated with long-term psychological distress and increased vulnerability to mental disorders. Eastwood et al. (2021) demonstrated that young transgender women from marginalized racial and ethnic backgrounds were more likely to have been sexually abused in childhood, contributing significantly to higher rates of homelessness, depression, and PTSD.

For transgender women of color, these risks are exacerbated by systemic inequalities, including limited access to healthcare and economic insecurity (Arayasirikul et al., 2022).

Finally, ACEs that involve discrimination and gender non-affirmation are associated with poorer general health and more days physically ill among transgender individuals through three indirect pathways: increased mental distress, discrimination, and gender non-affirmation, each of which further worsens physical health (Strenth et al., 2024).

#### 3.2.6. Intersectional Factors: Ethnicity, Socioeconomic Status, and Geography

Several studies included in this review show that exposure to ACEs and its consequences in TGNC populations are not uniform, but are strongly influenced by intersecting factors such as ethnicity, socioeconomic conditions, and cultural–geographical contexts.

In terms of race and ethnicity, Eastwood et al. (2021) show that young transgender women from marginalized racial and ethnic backgrounds are more likely to have been sexually abused in childhood, which contributes significantly to higher rates of homelessness, depression, and PTSD. Similarly, Ricks and Horan’s (2023) findings indicate that for Black transgender women, the protective effects of gender affirmation and social support may be undermined when these women are simultaneously exposed to intimate partner violence, highlighting how ethnicity and gender identity interact to shape vulnerability. Socioeconomic disadvantage is also a recurring theme in the literature reviewed. Discriminatory hiring practices and exclusion from stable employment reported in several studies (e.g., Ricks & Horan, 2023; Sizemore et al., 2022; Suarez et al., 2021) force many TGNC individuals into precarious or insecure employment, including sex work, which in turn increases vulnerability to violence and exploitation. This economic marginalization also limits access to resources that could mitigate the effects of ACEs, such as safe housing, healthcare, and social support networks.

Geographical and cultural contexts play a role in the manifestation of ACEs. For example, studies conducted in China report that family stressors—including non-acceptance, marriage pressure, and reproductive expectations—are associated with family violence and suppression of gender expression (Chan et al., 2024). These culturally specific dynamics highlight how, outside of Western contexts, traditional and TGNC-specific ACEs are often intertwined with normative familial and community obligations, which may impact both the nature of adversity experienced and the likelihood of disclosure.

Overall, the studies reviewed suggest that intersectional dimensions such as ethnicity, class, and geography influence three critical aspects: (a) exposure to ACEs, which is heightened in marginalized groups; (b) coping strategies, which may be constrained by poverty and social isolation; and (c) access to gender- and trauma-informed care, which is more limited for TGNC individuals living in disadvantaged socioeconomic circumstances or in cultural contexts with fewer supportive services.

This thematic reorganization highlights the interplay between traditional and TGNC-specific ACEs and how common forms of adversity and identity-based stressors come together to influence mental health trajectories in TGNC populations.

## 4. Discussion

This systematic review examined the association between ACEs and mental health outcomes in transgender and gender nonconforming individuals based on an analysis of recent scientific literature. Compared to previous reviews conducted prior to 2020—which already emphasized the increased risk of adverse childhood experiences (ACEs) in TGNC populations (e.g., Tobin & Delaney, 2019)—the emerging body of research from 2020 to 2024 shows an increasing emphasis on the nuances of nonbinary experiences and highlights the urgent need for more specific tools.

Initially, 6124 articles were identified through database searches. After removing duplicates and applying exclusion criteria, 4639 entries were screened for relevance. Following a rigorous screening process, 279 full-text articles were screened for eligibility, which ultimately led to the inclusion of 42 studies that met all criteria. These studies focused on different areas, including the following:

- Types of ACEs experienced by TGNC individuals, such as childhood maltreatment, family rejection, and peer victimization.
- Mental health consequences of ACEs, including increased risk for depression, anxiety, PTSD, suicidality, and substance use.
- Protective factors and resilience mechanisms, such as social support, gender affirmation, and access to mental healthcare.
- Implications for clinical interventions, including trauma-informed and gender-specific treatment strategies.

The synthesis of these findings can be usefully interpreted through the lens of the Minority Stress Model, which provides a conceptual framework for understanding how distal stressors (such as structural discrimination, systemic barriers, and community-level violence) and proximal stressors (such as family rejection, peer victimization, and internalized stigma) interact with traditional forms of childhood adversity. This model also helps to clarify the cumulative and interacting pathways that lead from early adverse experiences to negative mental health outcomes, while highlighting the potentially buffering role of resilience factors such as gender-affirming caregiving, social support, and community connectedness. Specifically, the results of this research show that TGNC-specific ACEs—rooted in stigma and identity-based rejection—pose an additional burden beyond traditional ACEs and exacerbate the stress processes of minorities across the lifespan.

Studies consistently indicate that traditional forms of childhood maltreatment—emotional, physical, and sexual abuse, neglect, and other forms of family dysfunction—are significantly more prevalent among TGNC individuals than cisgender individuals (Biedermann et al., 2021; Feil et al., 2023). This is consistent with previous research showing that transgender individuals are more likely to experience family rejection, school victimization, and community violence, all of which contribute to cumulative traumatization over the life course (Cao et al., 2023; Garthe et al., 2022). The clinical significance of these findings is clear: interventions must include early trauma assessment in TGNC populations, as these traditional ACEs alone are a strong predictor of poor mental health outcomes such as depression, PTSD, and suicidality.

In addition to traditional ACEs, the study also emphasizes forms of adversity that are solely related to gender identity. Transphobia-specific ACEs, such as forced gender conformity, denial of gender identity, targeted discrimination, and rejection, represent a separate trauma category (Austin et al., 2022; Arayasirikul et al., 2022). These experiences intensify the stress processes of minorities and add an additional layer of vulnerability. Findings indicate that these specific adversities impact mental health by fostering internalized stigma and social withdrawal. In addition, some studies indicate that the online environment has become a new context for this form of disadvantage: the reduction of online protection can promote cyber-victimization, harassment, and the dissemination of discriminatory content, thereby increasing anxiety, depression, and isolation (Lian et al., 2022). The use of sexual harassment as a punitive response to gender nonconformity (M. A. Price et al., 2023) reflects broader societal mechanisms of social control that have become harmful in modern contexts. The presence of these TGNC-specific adversities underscores the need for gender-specific interventions that go beyond general trauma-informed care.

The results also suggest that the impact of ACEs is exacerbated by intersectional factors such as ethnicity, socioeconomic status, and cultural–geographical context. For example, Eastwood et al. (2021) found that young transgender women from marginalized racial and ethnic backgrounds were more likely to have been sexually abused in childhood, which contributed significantly to higher rates of homelessness, depression, and PTSD. Similarly, Ricks and Horan (2023) demonstrated that Black transgender women are disproportionately affected by intimate partner violence, reducing the protective effect of gender identity. Socioeconomic disadvantage exacerbates this vulnerability: exclusion from stable employment and discriminatory hiring practices lead to economic insecurity, which increases the risk of exploitation (Ricks & Horan, 2023; Sizemore et al., 2022; Suarez et al., 2021). These conditions limit access to resources such as safe housing and healthcare. Geography also plays a role: in China, family stressors—non-acceptance, marriage pressure, and reproductive expectations—are associated with family violence and repression of gender expression (Chan et al., 2024). Intersectionality must be considered in interventions, particularly in policy design, as exposure to ACEs, coping resources, and access to care are all shaped by these intersecting axes of marginalization.

Despite the severe psychological impact of ACEs, this review also provides evidence of protective factors. Social support, community engagement, and a gender-equitable environment reduce psychological distress (Chakrapani et al., 2022; Ricks & Horan, 2023; Thomeer et al., 2022). The presence of a single affirming adult significantly reduces suicidality (Marquez-Velarde et al., 2023), while access to gender affirmation (e.g., use of pronouns, hormone therapy) is associated with better mental health (Ramos & Marr, 2023; Feil et al., 2023). These findings emphasize that protective resources are modifiable factors: increased access to affirming care and supportive networks is a critical prevention strategy. Nonbinary individuals appear to be more likely to experience peer victimization and social rejection than binary transgender individuals (Strauss et al., 2020; Klemmer et al., 2021). These specific findings suggest that intervention strategies need to be sensitive to gender diversity and not just designed for binary identities.

Overall, the reviewed findings emphasize that the combined effects of traditional and TGNC-specific ACEs can be coherently understood within the framework of the Minority Stress Model: structural and interpersonal adversities interact over time to shape developmental trajectories and vulnerability to mental health problems, while protective factors act as moderators within the same framework. This integrative perspective forms a conceptual bridge between the empirical findings and the recommendations for clinical practice presented below. Although this review shows clear patterns in the included studies, it is important to critically consider the methodological characteristics of the evidence base when interpreting these results. The field continues to be dominated by cross-sectional and self-reported studies, which, while valuable for capturing prevalence and associations, inherently limit causal conclusions and the generalizability of results. These methodological limitations suggest that the strength of evidence for identifying patterns of co-occurrence (e.g., of ACEs and mental health outcomes) is greater than for conclusions about temporal sequences or mechanisms.

In interpreting the results of this review, we considered the methodological quality of the included studies, which was assessed using the JBI critical appraisal tools. As the vast majority of studies were rated as moderate to high quality, the thematic synthesis predominantly reflects findings derived from methodologically sound sources. When results were consistent across multiple studies with higher quality ratings, they were interpreted with greater confidence. In contrast, the few results that came from individual studies—particularly those of lower methodological quality—were treated as tendentious or exploratory. This approach strengthens the reliability of the conclusions drawn and highlights areas where further research may be needed.

In summary, although the overall methodological quality of the included studies was mostly moderate to high, the synthesis should be interpreted with these structural limitations in mind. Stronger conclusions can be drawn where the results of several high-quality studies converge, while other observations—particularly those derived from single studies or from qualitative reports—should be considered exploratory. Distinguishing between evidence-based conclusions and more speculative hypotheses enhances the credibility of the review and clarifies priorities for future research.

## 5. Clinical Implications

The intersection of transgender and gender nonconforming identities and adverse childhood experiences has profound implications for clinical practice and requires a holistic, trauma-informed, and affirming approach to care. Clinical providers must recognize the increased risk of mental health issues among TGNC individuals due to systemic discrimination, stigma, and the compounding effects of ACEs. Addressing these challenges requires creating an environment that prioritizes safety, inclusivity, and affirmation of gender identity (Feil et al., 2023).

### 5.1. Trauma-Informed Care

An important strategy is the integration of a trauma-informed care approach, which focuses on understanding the far-reaching effects of trauma, recognizing signs of trauma in patients, and incorporating this awareness into all aspects of treatment (Biedermann et al., 2021). This approach can reduce re-traumatization, especially in healthcare settings where TGNC individuals often face microaggressions or devaluation of their identity. A trauma-informed care approach recognizes that many aspects of psychological distress are related to early trauma, systemic marginalization, and discrimination. Therefore, it is important to examine the role of trauma in identity formation and interpersonal relationships without pathologizing gender identity. Ensuring a safe, gender-affirming environment free of negative stereotypes is essential, as TGNC individuals already face discrimination in other areas of healthcare. Supporting self-integration and processing traumatic memories while validating their subjective experience of gender identity is essential.

### 5.2. Affirmative Practices

Gender affirmation is a cornerstone of effective clinical interventions for TGNC individuals. Studies show that gender affirmation, including the use of correct names and pronouns and facilitating access to gender-affirming healthcare, significantly reduces mental health disparities (M. N. Price & Green, 2023; Ramos & Marr, 2023). Clinicians should actively engage in creating a positive environment that can mitigate the negative mental health effects associated with social rejection and internalized transphobia (Domínguez-Martínez et al., 2023). Training programs for healthcare providers on cultural competency and the unique needs of TGNC populations are essential to promoting positive practices. Interventions that address minority stress and the internalization of stigma have an impact on mental well-being. It is important to help people recognize and deconstruct internalized stigma in order to promote a more positive self-construction. Validating and affirming gender identity can help to reduce gender dysphoria and improve psychological well-being. In addition, integrating empowerment practices by supporting strategies to overcome discrimination and build resilience is essential.

### 5.3. Intersectional Considerations

Clinical interventions should take an intersectional view and recognize how ethnicity, socioeconomic status, and other social determinants of health impact the experiences of TGNC individuals. For example, Black transgender women often face complex challenges due to racial and gender discrimination that require culturally sensitive and equity-focused approaches to care (Ricks & Horan, 2023). Clinicians need to assess and address these intersecting vulnerabilities in order to provide comprehensive support. Interventions to foster relationships and build support networks are crucial, as social isolation and lack of family support are significant risk factors. Strengthening social support networks by connecting people to inclusive TGNC communities is essential. This includes offering family support interventions and psychoeducation to reduce the likelihood of rejection and humiliation. In addition, working on relationship dynamics, particularly experiences of rejection, abandonment, and marginalization, is important.

### 5.4. Early Screening and Intervention

Early detection of ACEs and associated risks is critical. Routine screening for trauma and mental illness in TGNC populations can help clinicians proactively intervene. Instruments to assess ACEs and gender-specific challenges should be integrated into the clinical setting to create individualized care plans (Feil et al., 2023; M. A. Price et al., 2023; Suarez et al., 2021; Tran et al., 2023).

Several studies included in this systematic review used existing screening instruments to assess ACEs, such as the Adverse Childhood Experience Questionnaire (Felitti et al., 1998), the Revised Childhood Abuse Questionnaire (Spielmann et al., 2022), or the Composite Abuse Scale (Revised)—Short Form (CASR-SF; Ford-Gilboe et al., 2016). However, the majority of the studies analyzed opted to develop ad hoc questions specifically designed to investigate the variables of interest related to adverse childhood experiences.

M. A. Price et al. (2023) highlight that standard measures of ACEs may not fully capture the specific experiences of this population, particularly in relation to gender. In their study, the authors aimed to better understand the specificity of gender-specific adversities and their relationship to standard ACE measures. For example, the Expanded ACEs Scale (Karatekin & Hill, 2019) was found to have some limitations in relation to the specific experiences of transgender and gender diverse individuals (the items on discrimination and social isolation do not sufficiently address gender discrimination and forms of active rejection based on gender identity). Similarly, the Gender Minority Stress and Resilience (GMSR) scale (Testa et al., 2015), while assessing the stress of gender minorities, does not appear to analyze in depth the range of traumatic experiences of transgender and gender diverse youth, such as physical and sexual violence related to gender identity (M. A. Price et al., 2023). Arayasirikul et al. (2022) and Strauss et al. (2020) also conducted in-depth studies in which they examined experiences of abuse before the age of 16 in relation to gender identity and explicitly asked participants whether the abuse they experienced was because of their transgender identity.

In addition, characteristics of emotion dysregulation should be assessed (Cao et al., 2023). Given the high prevalence of anxiety, depression, PTSD, and suicidal ideation in TGNC individuals with a history of ACE, targeted mental health interventions are critical. Therapists should consider the three-phase model of therapeutic approach to trauma treatment: Establishing Safety, Processing the Trauma, Integrating the Self, and Promoting Social Reconnection and Personal Growth (Biedermann et al., 2021). It is also important to focus treatment on promoting a secure state of mind in relation to attachment (Cussino et al., 2021; Kozlowska et al., 2021; Sizemore et al., 2022). Given the high prevalence of complex trauma, specific interventions are required. Techniques such as mindfulness-based interventions (MBI), compassion-focused therapy (CFT), and acceptance and commitment therapy (ACT) can reduce hypervigilance and dissociative symptoms. Trauma-sensitive approaches such as EMDR and dialectical behavioral therapy (DBT) can facilitate the processing of traumatic experiences and at the same time manage the stress triggered by traumatic memories. In addition, teaching strategies for coping with gender dysphoria and alleviating the stress associated with body incongruence—such as the principles of sensorimotor psychotherapy—can be beneficial. In addition, peer support programs and community-based interventions can provide a safe space for TGNC individuals to share experiences and build resilience.

### 5.5. Family Education Programs

As transgender youth often face rejection and violence in their family environment, it is critical to establish educational programs aimed at guiding families through the acceptance process (Brangwin et al., 2023; Domínguez-Martínez et al., 2023). These initiatives should also encourage families to provide meaningful support to their TGNC relatives. For adolescents, family-oriented interventions aimed at reducing rejection and promoting acceptance can have long-term protective effects on mental health (Gamio Cuervo et al., 2022). In particular, strengthening support networks is critical to helping individuals build connections to inclusive TGNC communities. In addition, family support programs and interventions that address romantic relationships, along with psychoeducational initiatives, can help reduce the risk of rejection and humiliation. Clinical work should also focus on relationship dynamics, paying particular attention to experiences of rejection, abandonment, and exclusion from social belonging. Creating safe spaces for dialogue between family members and TGNC individuals can lead to greater understanding and acceptance, promote supportive relationships, and reduce the psychological distress associated with social isolation.

### 5.6. Policy and Advocacy

Clinical practice must extend beyond individual patient care to include advocacy for systemic change. This includes supporting policies to protect TGNC rights, improve access to gender-specific care, and address the social determinants of health more broadly (Wichaidit et al., 2021). Collaboration with stakeholders and policy makers can enhance efforts to reduce barriers that perpetuate health inequalities among TGNC individuals (Domínguez-Martínez et al., 2023; M. A. Price et al., 2023). Therapeutic work cannot ignore a critical understanding of the social context that perpetuates discrimination and vulnerability in minority populations. Clinicians should be aware of the impact of systemic discrimination, economic instability, and barriers to accessing healthcare. It is critical that they support the efforts of those affected by directing them to legal, medical, and social resources that include TGNC. Working with professionals who have specialized knowledge of TGNC issues ensures a multidisciplinary and integrated approach to treatment.

### 5.7. Holistic Approaches to Care

Holistic approaches that address both physical and mental health are essential. Access to gender reassignment surgery and hormone therapies should be considered an integral part of care, not the only one. In addition, addressing issues such as housing insecurity, substance use, and workplace discrimination can further promote recovery and resilience. Clinicians should also consider implementing multidisciplinary approaches that involve collaboration between primary care providers, mental health specialists, and social workers. These teams can work together to meet the unique needs of TGNC individuals and ensure that care is comprehensive and affirming (Strauss et al., 2019). Clinical work needs to be personalized and respectful of individual characteristics without reducing a person’s experience to a single, stereotypical transgender narrative. It is important to avoid normative or pathologizing approaches while acknowledging the diversity of gender identities and experiences. It is important to support individuals in making fully informed and conscious decisions about their journey and avoid pigeonholing them. Integrating gender narrative exploration into therapy allows for an authentic self-discovery process that is free from external pressures. Clinicians should support self-exploration without reinforcing stereotypes and ensure that individuals have the time they need to consolidate their identity and make considered choices.

### 5.8. Education and Training

Ongoing education and training of healthcare providers is critical to improving care for TGNC individuals. Healthcare providers should be educated about the unique needs and vulnerabilities of TGNC populations and empowered to provide affirming and compassionate care (Tran et al., 2023). Training should also include strategies to reduce implicit bias and promote a welcoming environment for all patients. In addition, training programs should emphasize the importance of personalized interventions and ensure that clinical approaches respect the diversity of gender identities without reinforcing normative or pathologizing frameworks. Providers should be empowered to support informed decision-making, avoid gatekeeping practices, and facilitate gender exploration in a non-prescriptive manner. In addition, promoting awareness of the sociocultural context—including systemic discrimination, economic precarity, and barriers to accessing care—can enhance clinicians’ ability to advocate for TGNC-inclusive resources and collaborate with interdisciplinary teams to provide holistic and integrated care.

### 5.9. Policy Implications

From a policy perspective, the findings of this review strongly support the implementation of gender-affirming frameworks as outlined in the WPATH Standards of Care Version 8 (SOC-8; Coleman et al., 2022). These international guidelines provide a structured, evidence-based approach to improving the well-being of TGNC individuals, particularly during childhood and adolescence—the critical periods when adverse experiences are most likely to occur. The SOC-8 emphasizes the importance of early recognition, affirmation, and support of gender identities in family, school, and healthcare settings (see Section 6 and Section 7), providing a direct response to some of the most commonly reported ACEs in this population, including rejection in the family, victimization in school, and psychological devaluation. In addition, the SOC-8 advocates for the creation of safe and inclusive environments in schools and services by promoting the use of chosen names and pronouns, training for professionals, and active protection from bullying and misgendering. These recommendations align with our findings on the critical role of peer victimization and institutional discrimination in the development of long-term mental health problems. The guidelines also call for the removal of systemic barriers to care, such as restrictive access criteria or pathologizing protocols, which our research shows exacerbate secondary stressors with long-lasting consequences.

By embedding reforms in public health, education, and social services based on these internationally recognized standards, policymakers can better address the structural causes of trauma and inequality. The gap between the life experiences of TGNC individuals and the institutional settings in which they must navigate remains a central source of problems. Bridging this gap through policies aligned with SOC-8 offers a concrete opportunity to reduce exposure to identity-based ACEs and mitigate their long-term effects.

## 6. Limitations and Future Directions

In addition to the study-level limitations listed below, it should be noted that the overall evidence base remains geographically concentrated (with most studies from North America and Europe) and methodologically heterogeneous, factors that limit the generalizability and comparability of the results.

The authors of the studies examined point to several methodological limitations. One of these is the use of self-report instruments, which can lead to bias due to social desirability effects (Ybarra et al., 2022; Tran et al., 2023). In addition, many studies were cross-sectional, which limits the causal interpretation of the relationship between ACE and psychological outcomes (Thoma et al., 2021; Xu et al., 2023). Future research should prioritize longitudinal studies to establish clearer associations and better determine the long-term psychological effects of ACEs (Wichaidit et al., 2021). A commonly reported limitation in studies is the lack of distinction between binary and nonbinary transgender individuals, with many studies grouping them together despite evidence that nonbinary individuals may have different vulnerabilities and coping mechanisms (Thomeer et al., 2022; Gamio Cuervo et al., 2022). Future studies should incorporate gender-specific methods that take into account the unique experiences of different subgroups within the TGNC population. Another key issue is the inconsistent use of clinical assessment tools to measure PTSD, depression, and suicidality, which has led to difficulties in comparing the results of different studies (Tubman et al., 2024). To improve the reliability of research, standardized and validated measurement instruments should be used. In addition, studies have often not fully explored the cumulative effects of multiple traumatic experiences and their role in maintaining cycles of vulnerability and re-victimization (Xu et al., 2023; Suarez et al., 2021). Future research should investigate how early traumatic experiences affect later exposure to violence and discrimination and the role of protective factors in interrupting these cycles. Finally, there is an urgent need for clinically informed intervention strategies that integrate trauma-informed and gender-specific approaches to care. Many existing interventions do not adequately address the intersection of trauma, identity-based stressors, and systemic discrimination faced by TGNC individuals (Biedermann et al., 2021; Ricks & Horan, 2023). Future research should work to develop and evaluate tailored clinical interventions that specifically address the complex needs of this population.

### Persistent Gaps in the Literature

Despite the growing body of research, there are still gaps that require further attention. Most studies continue to be based on random sampling, which limits the representativeness of the results. There is a distinct lack of longitudinal studies capable of tracking the long-term effects of ACEs on the mental health of TGNC individuals. In addition, the geographical concentration of the evidence base—predominantly in North America and Western Europe—raises concerns about its global applicability. Studies from underrepresented regions remain scarce, leaving the impact of sociocultural differences largely unexplored. Finally, few studies have systematically examined the combined effects of intersecting marginalizations (e.g., racism, poverty, migration status) that could significantly influence exposure to ACEs and access to protective resources.

## 7. Conclusions

The findings of this review highlight the significant impact of ACEs on the mental health of TGNC individuals and emphasize the need for trauma-informed, gender-affirming, and intersectional approaches to care. In addition to individual-level interventions, systemic changes are essential to address the structural inequities that exacerbate the plight of TGNC populations caused by ACEs. Policy efforts should focus on expanding legal protections, ensuring equal access to healthcare, and promoting inclusive social environments that affirm diverse gender identities. Future research should continue to explore the nuanced ways in which ACEs impact the mental health of TGNCs, as well as the most effective strategies for intervention and support.

## Figures and Tables

**Table 1:** Search strategy: keywords used in the 4 databases.

“gender dysphoria” or “gender identity disorder” or “transsexualism” or “transgender” or “gender identity” or “transsexual” or “transsexualism” or “gender variant” or “transgender” or “transgenderism” or “gender variant” or “gender variance” or “atypical gender identity” or “cross sex” or “cross-gender” or “gender incongruence” or “gender reassignment” or “gender reaffirmation surgery” or “gender transition” or “gender atypicality” or “non-conformed gender” or “reassignment surgery” or “sexual reassignment”
AND
“dissociative” or “dissociation” or “trauma” or “abuse” or “neglect” or “maltreatment” or “posttraumatic” or “ptsd” or “disorganized attachment” or “violence”

**Table 2:** Main studies exploring trauma and adverse experiences during childhood and adolescence.

Author, Year, Country	Sample Characteristics		Type of ACEs or Trauma	Study Design and Methodological Approach	Health Impacts and Clinical Implications
	Sample Size and Participants’ Age	Sex Assigned at Birth			
Arayasirikul et al. (2022) United States	*N* = 300 Age = 16–24 years	AMAB	Transphobia Verbal abuse Physical abuse Sexual abuse	Quantitative Cross-sectional	Exposure to high levels of ACEs is detrimental to mental and sexual health and is associated with unsafe sexual practices.
Austin et al. (2022) United States–Canada	*N* = 372 Age = 14–18 years M. Age = 15.99 (1.232) years	AMAB AFAB	Transphobia Rejection Verbal abuse Physical abuse Emotional neglect Internalized self-stigma Microaggressions	Quantitative Cross-sectional	Interpersonal identity-based microaggressions are significantly associated with suicide attempts, but not ideation. Emotional neglect by family, school belonging, and internalized self-stigma are significantly related to past 6-month suicidality. Feeling disconnected from school is associated with higher past 6-month suicidality. Internalized self-stigma is related to suicidal ideation. Early interventions with caregivers are essential to fostering supportive attitudes toward gender diversity. It is important to provide caregivers with updated information on the importance of adopting an affirming approach; what affirmative parenting looks and sounds like; and the harmful effects of rejecting, invalidating, or minimizing a child’s transgender identity.
Barboza-Salerno and Meshelemiah (2024) United States	*N* = 1368 (N = 274 transgender and N = 1162 cisgender) Age = 18–72 years M. Age = 54.83 (15.2) years	AMAB AFAB TGNB	Household mental illness Household intimate partner violence Emotional abuse Physical abuse Sexual abuse Parental divorce or separation Household substance use Living with an incarcerated household member	Quantitative Cross-sectional	Gender minorities have higher LSA risk and more ACEs, with depressive symptoms mediating this link. Transgender groups face up to eight times higher LSA risk, showing non-linear risk patterns. Depressive symptoms have a stronger mediating effect for transgender people; alcohol use and social support were not mediators.
Belloir et al. (2023) United States	*N* = 166 (99 AFAB + 67 AMAB) Age = 21–74 years M. Age = 38.6 (12.2) years	AMAB AFAB	ACE	Quantitative Multi-site longitudinal study	ACEs are an important predictor of psychological distress. General self-efficacy partially mediates the positive association between ACEs and psychological distress. Increasing general self-efficacy in gender minorities may have therapeutic benefits for mental health. Early screening of gender minority youth can help identify potential abuse or neglect, enabling timely interventions like psychological first aid or cognitive behavioral therapy to prevent further adverse experiences.
Biedermann et al. (2021) Germany	*N* = 187 (94 AMAB + 93 AFAB) M. Age AMAB = 40.9 (13.0) years M. Age AFAB =30.9 (10.9) years	AMAB AFAB	Emotional abuse Physical abuse Sexual abuse Emotional neglect Physical neglect Parents showing violent behavior Bullying Forced to behave according to sex	Quantitative Cross-sectional	Association of ACEs with adult depression and suicidality. Time since the beginning of hormone therapy does not influence adult depressive symptoms or suicidality. In light of the high prevalence and the associated psychological burden, ACEs should be assessed regularly in trans people, especially when clinical symptoms suggest it.
Brangwin et al. (2023) United States	*N* = 121,150 (60,973 female + 57,873 male + 203 T AMAB + 175 T AFAB + 344 T do not identify + 1052 gender not sure) Age = 11–19 years M. Age = 14.74 (1.78) years	AMAB AFAB	Family physical abuse	Quantitative Cross-sectional	GM adolescents experience higher levels of family physical abuse and remain at greater overall risk for suicide attempts compared to cisgender adolescents. No significant differences are found within gender minority groups, indicating that all GMY face similarly high levels of family physical abuse. Adolescents with multiple minority identities (e.g., GM, sexual minority, female) report higher levels of family physical abuse than those with fewer or no minority identities. Family physical abuse is linked to increased odds of suicidal behavior across all gender identity groups. The strength of the association between family physical abuse and suicide attempts is similar for both cisgender and gender minority adolescents.
Cao et al. (2023) China	*N* = 971 (296 AFAB + 675 AMAB) M. Age = 24.5 (6.4) years	AMAB AFAB	Emotional abuse Physical abuse Sexual abuse Emotional neglect Physical neglect	Quantitative Cross-sectional cohort study	NSSI behaviors in transgender individuals are linked to childhood abuse and emotional dysregulation traits. Emotional dysregulation partially mediates the connection between childhood abuse and NSSI behaviors. Screening for emotional dysregulation traits and providing timely interventions are crucial. Efforts should focus on improving the environment for transgender people to reduce childhood abuse. Reducing childhood abuse may decrease NSSI behaviors and extreme events in the transgender population.
Chakrapani et al. (2022) India	*N* = 27 M. Age = 25 (3.0) years	AFAB	Stigma Discrimination Violence in diverse settings/structural violence (family, schools, workplace) Bullying	Qualitative	Findings reveal the impacts of structural violence. Fear of discrimination and anticipated stigma can lead individuals to conceal their gender identity or expression, resulting in negative mental health outcomes. Family (non)acceptance is linked to mental health. Heavy alcohol use or smoking are coping strategies that negatively impact health and may be associated with masculinity or stress coping. Participants with family or friend support do not engage in such behaviors.
Chan et al. (2024) China	*N* = 1063 Age = 18–30+ years	AMAB AFAB	Gender identity and expression change efforts Physical abuse Emotional abuse Sexual abuse Financial abuse	Quantitative	76% of TNB individuals reported having encountered at least one form of violence perpetrated by their family members. Transfeminine individuals reported experiencing emotional and physical abuse; transmasculine individuals were more likely to be subjected to gender identity and/or expression change efforts.
Cussino et al. (2021) Italy	*N* = 52 (25 AFAB + 27 AMAB) Age = 17–52 years M. Age = 31.33 (10.46) years	AMAB AFAB	Prostitution Violence by the father/mother Father/mother alcoholics Death of mother/father/brother/sister Rejection by mother/father Neglect by mother/father	Quantitative Cross-sectional	A total of 92.3% of participants have an insecure attachment state of mind. Among those with GD, 71.2% exhibit a dismissing attachment state, while 21.2% have an entangled state. Half of the sample reports one or more traumatic life events. AAI data reveal frequent unresolved trauma among those with insecure attachment. No traumatic experiences related to attachment are reported by securely attached participants. Findings suggest that interventions for transgender patients should focus on promoting a secure state of mind regarding attachment and traumatic experiences.
Domínguez-Martínez et al. (2023) Mexico	*N* = 245 (198 AMAB + 47 AFAB) Age = 18–65 years M. Age = 30.9 (10.2) years	AMAB AFAB	Physical violence Psychological violence Sexual violence Family rejection Peer rejection	Qualitative Descriptive study Retrospective research interviews	Transgender people face a high risk of various forms of violence during adolescence, with psychological violence being the most common, followed by physical and sexual violence. Transgender people are at high risk of social rejection from an early age across various contexts. Rejection, seeking recognition of gender identity, and requesting to be called by their chosen name are the strongest predictors of all types of violence.
Eastwood et al. (2021) United States	*N* = 102 Age = 18–24 years M. Age = 22.30 (1.62) years	AMAB	Childhood sexual abuse Forced or coerced sexual contact Housing instability and homelessness Poverty and food insecurity Social marginalization and discrimination Engagement in high-risk survival behaviors (e.g., sex work, drug use) Lack of social support from family and transgender peers	Quantitative Cross-sectional	A total of 75.5% of young transgender women of color experienced homelessness, with 92.9% living in poverty and 50% relying on sex work. Mental health distress was widespread, with 51.3% screening positive for depression, while only 26.5% achieved HIV viral suppression and 12.7% maintained consistent HIV care. Childhood sexual abuse was strongly linked to homelessness, high-risk behaviors, and poor health outcomes. Trauma exposure, economic instability, and lack of support create a syndemic effect, exacerbating health disparities and underscoring the need for integrated housing, mental health, and HIV care interventions.
Feil et al. (2023) Austria	N = 70 (35 GNC + 35 cisgender) GNC = 35 (18 AFAB + 12 AMAB + 5 nonbinary) M. Age = 29.5 (2.2) years	AMAB AFAB	Emotional abuse Physical abuse Neglect Witnessing violence Peer abuse/bullying Sexual violence	Quantitative Cross-sectional	TGD people are significantly more likely to experience ACEs than cisgender people (parental and peer abuse are especially common). Sexual abuse is not more prevalent. TGD individuals report an earlier onset of ACEs at 5 years of age and a higher prevalence of depression, PTSD symptoms, and anxiety. There is an urgent need to prevent childhood trauma and foster resilience. Healthcare providers should assess ACEs in TGD people and ensure a safe, affirming care environment.
Gamio Cuervo et al. (2022) Unites States	*N* = 12 Age = 23–32 years	AMAB AFAB	Family rejection Intergenerational violence	Qualitative	Family rejection in Latinx families is connected to the cycle of family violence and intergenerational trauma and is a manifestation of cumulative trauma that influences decisions. Understanding cultural values in individual and family environments is crucial to understanding rejection. These factors may increase the need for Latinx trans children to negotiate or distance themselves relationally.
Garthe et al. (2022) United States	*N* = 4464 (1116 male + 1116 female + 1116 T + 1116 GE) (Students in grades 8 to 12)	AMAB AFAB	Peer victimization Dating violence	Quantitative Cross-sectional	Transgender and gender-expansive youth face an alarming risk of suicidal ideation, highlighting the urgent need for suicide prevention efforts. Suicidal ideation is mediated by peer victimization, dating violence, substance use problems, and depressive symptoms. Programs that promote mental health and prevent bullying, dating violence, substance use, and related problems are essential.
Garthe et al. (2023) United States	N = 4464 (1116 male + 1116 female + 1116 T + 1116 GE) (Students in grades 8 to 12)	AMAB AFAB	Peer victimization (physical, verbal, or cyber forms of bullying) Dating violence (physical or psychological abuse from dating partners)	Quantitative Cross-sectional	Peer victimization decreases as youth progress through high school among GNC youth. Transgender and gender-expansive youth experience more physical, verbal, and cyber victimization than their peers. Eighth- to tenth-grade gender-expansive youth report higher rates than twelfth graders. Eighth graders experience the highest levels of physical peer victimization; verbal victimization remained high across all grades. Cyber-victimization is higher among female, transgender, and gender-expansive youth in eighth grade. Transgender and gender-expansive youth report higher levels of physical and psychological dating violence.
Kaltiala and Ellonen (2022) Finland	*N* = 123,663 (790 opposite sex + 4351 nonbinary + 118,522 cisgender) Age = 14–20 years	AMAB AFAB	Sexual harassment (gender harassment, unwelcome sexual attention, and sexual coercion)	Quantitative Cross-sectional	GMY report higher rates of sexual harassment compared to cisgender youth. Both gender identity issues and sexual harassment are linked to mental health problems (e.g., emotional and behavioral disorders) Excessive experiences of sexual harassment among transgender adolescents are partially explained by confounding family-related and mental health factors and not exclusively by gender identity per se. Teaching coping skills and reducing internalized heterosexism can help alleviate minority stress. Interventions should prioritize identifying and treating mental health issues in gender minority youth. Addressing emotional and behavioral disorders can help reduce vulnerability to sexual harassment and improve overall well-being.
Klemmer et al. (2021) United States	*N* = 1496 (26 T + 717 cisgender boys + 753 cisgender girls) Age = 14–18 years M. Age = 16.0 years	AMAB AFAB	Bullying	Quantitative Cross-sectional	Socially assigned gender nonconforming adolescents face increased rates of bullying, school-related violence, and safety concerns compared to their gender-conforming peers. They are also more likely to miss school due to these safety concerns and experiences of bullying.
Kozlowska et al. (2021) Australia	*N* = 57 (24 AMAB + 33 AFAB) Age = 8.42–15.92 years M. Age = 12.96 (1.91) years	AMAB AFAB	Family conflict Loss via separation from a loved one or a close friend Bullying Maternal mental illness (most commonly depression) Paternal mental illness Financial stress Moving house that was stressful Domestic violence Maternal physical illness Physical abuse Sexual abuse Placement changes (foster care or between parents) Neglect Custody battle Intelligence	Quantitative Cross-sectional	Children with gender dysphoria are often in a context where multiple risk factors interact, including insecure attachment, unresolved loss or trauma, family conflict and disintegration, and exposure to various ACEs. GD in children is linked to developmental pathways involving insecure attachment, unresolved loss, and trauma. These pathways are shaped by family instability, ACEs such as maltreatment, and SES. A broad perspective is essential for understanding GD, considering its impact on distress, adaptation difficulties, multimorbidity, and overall well-being. Effective treatment requires a comprehensive biopsychosocial assessment of both the child and family. Therapeutic interventions should address the interconnected factors influencing the child’s experience. Efforts should focus on enhancing the child’s sense of acceptance and safety within family and peer relationships.
Lacombe-Duncan and Olawale (2022) Canada	*N* = 11 Age = 20–60 years	AMAB	Familial exclusion Bullying ACE	Qualitative	TW experience ACEs such as parental substance use, mental health issues, and lack of security. Some ACEs, which are directly linked to transgender and gender nonconformity stigma, such as abuse, neglect, homelessness, familial rejection, and bullying, contribute to internalized trans stigma.
Lian et al. (2022) United States	*N* = 728 (334 T + 394 not sure) T = 137 AFAB + 197 AMAB not sure = 196 AFAB + 198 AMAB Students in grades 9 to 12	AMAB AFAB	Bullying	Quantitative Cross-sectional	TGNC students are more vulnerable to bullying victimization (higher rates of traditional and electronic bullying) than cisgender peers. AMAB GNC adolescents are linked with higher bullying victimization, while AFAB GNC are not.
Lowry et al. (2020) United States	*N* = 6082	AMAB AFAB	Bullying	Quantitative Cross-sectional	GNC males are at a greater risk for victimization than GNC females. Substance use among GNC male students may be a response to the higher prevalence of violence victimization they experience. No association between GNC female students and substance use.
M. A. Price et al. (2023) United States	*N* = 49 (44 T + 5 nonbinary/ 11 AMAB + 38 AFAB) Age = 11–20 years M. Age = 15.53 (1.73) years	AMAB AFAB	Bullying ACE Gender-related adversity	Qualitative	The results emphasize the significance of gender-related challenges faced by TGD youth, including verbal abuse, threats or acts of physical and sexual assault, discrimination, lack of affirmation, and rejection. A comprehensive assessment of both gender-related and non-gender-related adversity is essential to understanding the unique mental health challenges faced by TGD youth, given the highly distressing impact of gender-related adversities.
M. N. Price and Green (2023) United States	N = 8221 (3610 T + 4611 nonbinary) Age = 13–24 years	AMAB AFAB	Rejection	Quantitative Cross-sectional	TGNB youth with at least one accepting adult have 33% lower odds of attempting suicide in the past year; peer acceptance also plays a comparable key role in reducing suicide attempts. Acceptance has a greater impact on those AMAB compared to those AFAB.
Marquez-Velarde et al. (2023) United States	N = 27,715 TNB Age = 18–65+ years	AMAB AFAB	Family rejection Physical violence by family members Expulsion from home due to gender identity Emotional rejection (loss of family relationships) Restriction of gender expression	Quantitative Cross-sectional	Cumulative ACEs significantly increase suicidality, with predicted probabilities of suicide ideation rising from 72% (no rejection) to 97% (all five rejection experiences), and suicide attempts rising from 35% to 75% when all forms of family rejection are experienced. These findings underscore the need for family-focused interventions and structural policy changes to mitigate the impact of early-life rejection on transgender mental health.
Marx et al. (2021) United States	*N* = 610 Age = 13–18 years	AMAB AFAB	Childhood sexual abuse Sexual harassment/victimization Gender-based violence	Quantitative Cross-sectional	Sexual victimization, prejudiced peer victimization, problematic substance use, and AFAB are significant predictors of sexual victimization among TGNC adolescents. Sexual victimization, prejudiced peer victimization, sexual harassment victimization, and problematic substance use are associated with increased suicidal ideation. Greater parental support and a sense of school belonging are associated with lower levels of suicidal ideation.
McBride and Neary (2021) Ireland	*N* = 13 Age = 15–24 years	AMAB AFAB	Structural vulnerability	Qualitative	Trans youth experience harm due to institutionalized cisnormativity that fosters insecurity and confusion about gender identity. Trans youth resist exclusion through activism, education, and policy advocacy, but these actions also expose them to further risks. Coming out, educating peers, and challenging school norms are survival strategies that also confront cisnormative power structures.
Newcomb et al. (2020) United States	*N* = 214 (128 AFAB + 86 AMAB) M. Age AFAB = 19.04 (3.18) years M. Age AMAB = 21.91 (3.25) years	AMAB AFAB	Childhood sexual abuse	Quantitative Cross-sectional	Over one-third of TW and nonbinary AMAB youth reported experiencing CSA; nearly one-third of TW experienced penetrative CSA. Nonbinary AMAB youth had the highest rates of lifetime trauma (82.1%), followed by nonbinary AFAB youth (78%), TW (72.4%), and TM (59.1%). CSA and other violent experiences contribute to depression, suicidality, substance use, and sexual risk behaviors. Individual and structural interventions are essential to prevent and mitigate these experiences for TGD youth.
Ricks and Horan (2023) United States	*N* = 138 Age = 18–65 M. Age = 30.8 (10.0) years	AMAB	Childhood sexual abuse	Quantitative Cross-sectional	CSA is a common lifetime experience (40.6% of participants) but does not diminish the significant association between social GA and mental health outcomes.
Sizemore et al. (2022) United States	*N* = 213 M. Age = 34.27 (11.67)	AFAB	Childhood sexual abuse	Quantitative Cross-sectional	In TW, depression acts as a mediator between CSA and substance use problems, including alcohol use disorder. Secure attachment reduces the impact of CSA on both depression symptoms and substance use issues.
Strauss et al. (2020) Australia	N = 859 (639 AFAB + 220 AMAB) Age = 14–25 M. Age = 19.37 (3.15) years	AMAB AFAB	Familial physical abuse Extrafamilial physical abuse Familial sexual abuse Extrafamilial sexual abuse Other familial abuse Abuse within an intimate relationship	Quantitative Cross-sectional	A total of 24.8% of participants report familial physical abuse, and 7.5% experience familial sexual abuse. All forms of abuse are linked to poor mental health, with varying risk levels. Familial physical abuse is associated with a fourfold increase in lifetime suicide attempts. The six forms of abuse studied are linked to self-harm, suicide, and psychiatric diagnoses. Most participants do not attribute their abuse to their TGD status. High prevalence of abuse among TGD youth requires clinician awareness; enhanced education and sensitivity among healthcare providers are essential.
Strauss et al. (2019) Australia	*N* = 859 (T + GNC) Age = 14–25 years	AMAB AFAB	Peer rejection Bullying precarious housing	Quantitative Cross-sectional	Of the participants, 89.0% experience peer rejection, 22.0% face unstable housing situations, 74.0% experience bullying, and 68.9% face discrimination. Poor mental health outcomes are most strongly linked to negative experiences, particularly unstable housing and educational challenges. Participants with a history of suicide attempts are nearly six times more likely to have faced housing issues, including homelessness. These results emphasize the urgent need for improved mental healthcare and targeted interventions.
Strenth et al. (2024) United States	*N* = 274	AMAB AFAB	Psychological abuse Physical abuse Sexual abuse Neglect Witness domestic violence (psychological) Witness domestic violence (physical) Mental illness Household death Household divorce Household criminal behavior Household drug use	Quantitative Cross-sectional	ACEs led to increased mental distress, which was linked to worse general health and more days physically ill. ACEs increased discrimination, which led to more mental distress and thus worse general health and more days physically ill. ACEs led to more discrimination and gender non-affirmation, increasing mental distress and leading to worse general health and more days physically ill.
Suarez et al. (2021) United States	*N* = 131 Age = 21–64 years	AFAB	Psychological abuse Physical abuse Sexual abuse Neglect Witness domestic violence (psychological) Witness domestic violence (physical) Mental illness Household death Household divorce Household criminal behavior Household drug use	Quantitative Cross-sectional	A total of 92% of TM adults experience ACEs. ACEs are strongly linked to mental health issues (those with 4+ ACEs have over five times the odds of depression and suicidality), victimization, worse health outcomes (increased obesity, raising risks for diabetes, heart attack, and stroke), and risky behaviors in adulthood.
Thoma et al. (2021) United States	*N* = 1836 (1055 T + 773 cisgender) Age = 14–18 M. Age = 15.9 (1.2)	AMAB AFAB	Physical abuse Emotional abuse Sexual abuse	Quantitative Cross-sectional	Transgender adolescents report more psychological, physical, and sexual abuse in childhood than heterosexual cisgender adolescents. AFAB transgender adolescents are more likely to report psychological abuse from parents or other adults. Transgender males have higher odds of psychological abuse compared to female cisgender adolescents, while nonbinary AFAB adolescents do not show the same elevated risk when accounting for other abuse types. Higher childhood abuse rates may contribute to disproportionate mental health challenges among transgender adolescents. Providers should focus on parent–adolescent relationships when treating AFAB transgender adolescents.
Thomeer et al. (2022) Unites States	*N* = 87 TGNB M. Age = 37.55 (18.64) years	AMAB AFAB	Rejection Sexual abuse	Qualitative	Rejection and experiences of violence are common during childhood. These findings offer valuable insights into the lived experiences of transgender and nonbinary individuals, emphasizing the need for supportive interventions across different life stages.
Tran et al. (2023) United States	*N* = 141,615 (556 GM + 141,059 cisgender) Age = 18–	AMAB AFAB	Physical abuse Emotional abuse Sexual abuse Household mental illness Household substance misuse Household domestic violence Incarcerated household member Parental separation or divorce	Quantitative Cross-sectional	GM adults report more emotional and physical abuse, as well as a higher overall prevalence of ACEs. Greater ACE exposure is linked to higher rates of frequent mental distress and lifetime depression diagnoses. ACEs disproportionately affect GM populations, emphasizing the need for prevention strategies. Routine pediatric ACE screening could help mitigate long-term mental health impacts, especially for GM youth. Mental health and primary care providers should receive education on transgender health to ensure inclusive and effective care.
Tubman et al. (2024) United States	*N* = 248 (172 AFAB + 76 AMAB) M. Age = 22.61 (3.06) years	AMAB AFAB	Sexual abuse	Quantitative Cross-sectional	Participants with a history of CSA experience more substance use, minority stress, and relational issues, show higher alcohol-related problems and negative consequences from substance use, and report greater daily discrimination. These findings highlight the importance of addressing the impacts of CSA in therapeutic interventions for transgender emerging adults.
van Beusekom et al. (2020) Netherlands	*N* = 2185 (1069 boys + 1116 girls) Age = 11–18 years M. Age = 15.13 (1.89)	AMAB AFAB	Bullying	Quantitative Cross-sectional	Adolescents with high gender nonconformity experience more peer victimization and homophobic name-calling than those with low gender nonconformity. Gender nonconformity elicits stronger peer victimization in boys than in girls and stronger victimization (both general and homophobic) among youth with high same-sex attraction. Findings highlight the need to promote acceptance of gender diversity and strengthen social support for gender-nonconforming youth. Age-appropriate education on gender and sexual diversity should be introduced before early adolescence to reduce victimization risks.
Wichaidit et al. (2021) Thailand	*N* = 421 AFAB + 334 AMAB Students in the general education system: Year 7 (Matthayom 1) Year 9 (Matthayom 3) Year 11(Matthayom 5) and the vocational education system: Vocational Certificate Year 2.	AMAB AFAB	Being threatened Severe physical violence Intimate partner violence Sexual violence	Quantitative Cross-sectional	One-third of transgender girls report experiencing sexual violence in the past year. Both transgender boys and girls are significantly more likely than cisgender girls to experience all types of violence. Transgender boys have the highest levels of depressive symptoms, suicidality, and alcohol consumption. Transgender girls have the highest prevalence of experiencing sexual violence in the past year. Suicidality is higher among transgender youth compared to cisgender youth, with transgender boys reporting the highest rates. Transgender boys have a higher prevalence of alcohol consumption than cisgender boys, while transgender girls have lower rates.
Xu et al. (2023) China	*N* = 247 Age = 18–61 years	AMAB	Childhood sexual abuse	Quantitative Cross-sectional	Respondents who experience CSA and IPV are more likely to report negative general health and suicidal thoughts. CSAs are strongly linked to suicidal ideation. Multivariate logistic analysis shows no significant difference in the odds of suicidality between participants exposed to CSAs and those who were not.
Ybarra et al. (2022) United States	*N* = 582 nonbinary + 329 transgender Age = 13–17 years M. Age = 14.8 (0.7) years	AMAB AFAB	Sexual violence	Quantitative Cross-sectional	GMY are more likely to report experiencing sexual violence but not more likely to report perpetrating it. Risk factors for perpetrating sexual violence vary across gender identities (cisgender, transgender, and nonbinary), which should be considered in prevention programs. Transgender youth exposed to spousal abuse are more likely to report perpetrating sexual violence, compared to those not exposed to such abuse. Nonbinary youth who had experienced sexual harassment are almost 3 times more likely to report perpetrating sexual violence than those who had not. Nonbinary youth exposed to violent or nonviolent pornography are more likely to report using sexual violence. Among nonbinary youth, past-year aggression is associated with increased likelihood of sexual violence perpetration.

AAI = Adult Attachment interview; ACE = adverse childhood experience; AFAB = assigned female at birth; AMAB = assigned male at birth; CSA = childhood sexual abuse; GA = gender affirmation; GE = gender-expansive; GD = gender dysphoria; GM = gender minority; GMY = gender minority youth; GNC = gender nonconforming; IPV = intimate partner violence; NSSI = non-suicidal self-injury; PTSD = posttraumatic stress disorder; SES = socioeconomic status; T = transgender; TGD = transgender gender diverse; TGNB = transgender gender nonbinary; TGNC = transgender gender nonconforming; TM = transgender man; TW = transgender woman.

## Data Availability

No new data were created or analyzed in this study. Data sharing is therefore not applicable to this article, as all information supporting the conclusions is contained within the manuscript.
